# Examining the relationship of acetaminophen use during pregnancy with early language development in children

**DOI:** 10.1038/s41390-023-02924-4

**Published:** 2023-12-11

**Authors:** Megan L. Woodbury, Patricia Cintora, Shukhan Ng, Pamela A. Hadley, Susan L. Schantz

**Affiliations:** 1 Neuroscience Program, University of Illinois at Urbana-Champaign, Urbana, IL USA; 2 Beckman Institute for Advanced Science and Technology, University of Illinois at Urbana-Champaign, Urbana, IL USA; 3 Present Address: College of Engineering, Northeastern University, Boston, MA USA; 4 Department of Speech and Hearing Science, University of Illinois at Urbana-Champaign, Urbana, IL USA; 5 Department of Comparative Biosciences, University of Illinois at Urbana-Champaign, Urbana, IL USA

## Abstract

**Background:**

Acetaminophen is the only analgesic considered safe for use throughout pregnancy. Recent studies suggest that use during pregnancy may be associated with poorer neurodevelopmental outcomes in children, but few have examined language development.

**Methods:**

The Illinois Kids Development Study is a prospective birth cohort in east-central Illinois. Between December 2013 and March 2020, 532 newborns were enrolled and had exposure data available. Participants reported the number of times they took acetaminophen six times across pregnancy. Language data were collected at 26.5–28.5 months using the MacArthur-Bates Communicative Development Inventories (CDI; *n* = 298), and 36–38 months using the Speech and Language Assessment Scale (SLAS; *n* = 254).

**Results:**

Taking more acetaminophen during the second or third trimester was associated with marginally smaller vocabularies and shorter utterance length (M3L) at 26.5–28.5 months. More acetaminophen use during the third trimester was also associated with increased odds of M3L scores ≤25th percentile in male children. More use during the second or third trimester was associated with lower SLAS scores at 36–38 months. Third trimester use was specifically related to lower SLAS scores in male children.

**Conclusions:**

Higher prenatal acetaminophen use during pregnancy may be associated with poorer early language development.

**Impact:**

- Taking more acetaminophen during pregnancy, particularly during the second and third trimesters, was associated with poorer scores on measures of language development when children were 26.5–28.5 and 36–38 months of age.
- Only male children had lower scores in analyses stratified by child sex.
- To our knowledge, this is the first study that has used a standardized measure of language development to assess the potential impact of prenatal exposure to acetaminophen on language development.
- This study adds to the growing body of literature suggesting that the potential impact of acetaminophen use during pregnancy on fetal neurodevelopment should be carefully evaluated.

## Introduction

Acetaminophen (paracetamol) is the most common drug ingredient in the United States and only analgesic considered safe to use throughout pregnancy.^1^ Both the American College of Obstetricians and Gynecologists^2^ and Society for Maternal-Fetal Medicine^3^ recommend acetaminophen as the first-line pharmacological intervention for pain and/or fever during pregnancy as other analgesics have been linked to problems in both the pregnant individual and fetus.^3^ Studies indicate 50–65% of pregnant women in North America and Europe report taking acetaminophen, or a medication containing it, at least once during pregnancy.^1,4–6^ Little is known about the safety of acetaminophen use during pregnancy due to the lack of clinical trials in pregnant women and very few reported cases of adverse effects in the developing child;^1,7,8^ however, acetaminophen can cross the placenta,^9–11^ and there has been increased interest in examining whether acetaminophen use during pregnancy could be related to child health outcomes. While some studies report no relationship between prenatal acetaminophen exposure and child health and development,^12–15^ other studies in animals and humans suggest it may be related to poorer neurodevelopmental outcomes.^8,16–30^ Multiple epidemiological studies indicate that prenatal acetaminophen exposure is associated with motor delays,^4,31,32^ attention problems,^6,33–41^ and behavioral problems.^4,34,38,42–45^ Early language development is predictive of later IQ, reading ability, and school success,^46,47^ yet only a few studies have investigated whether there is a relationship between prenatal acetaminophen use and language development. In the Swedish Environmental, Longitudinal, Mother and child, Asthma and Allergy Study, acetaminophen use early in pregnancy was related to delayed language development in female children at 30 months.^48^ In the Norwegian Mother and Child Cohort study, acetaminophen use during pregnancy was associated with poorer scores on the communication scale from the Ages and Stages Questionnaire (ASQ) at both 18-months^31^ and 3 years.^4,49^

The few studies that have assessed the association between prenatal acetaminophen use and language development have relied upon self-report measures of acetaminophen collected 1–3 times during pregnancy, leaving ample room for inaccuracies in memory and not allowing for accurate evaluation of whether the timing of exposure is important. Further research is needed to better-understand the potential for prenatal acetaminophen exposure to impact early language development. In the Illinois Kids Development Study (IKIDS) cohort, pregnant people were recruited early in pregnancy, and participants were interviewed about acetaminophen use in six discrete periods of pregnancy, leaving less time between interviews and allowing for a more accurate evaluation of acetaminophen use during each trimester. In the present study, the association of prenatal acetaminophen use with language development was evaluated at 26.5–28.5 months using the MacArthur-Bates Communicative Development Inventories: Words and Sentences (CDI)^50^ and at 36–38 months using the Speech and Language Assessment Scale (SLAS).^51^

## Methods

### Study cohort

IKIDS is a prospective pregnancy and birth cohort in east-central Illinois, United States originally designed to evaluate the relationship of gestational exposure to phthalates and phenols with neurodevelopment.^52^ Participants whose children were included in this analysis were recruited between December 2013 and March 2020 at two local obstetric clinics and gave birth at two local hospitals. Clinics gave brochures regarding the study to patients at their first prenatal visit. Patients completed a reply card indicating their interest in being contacted about participation. Interested individuals were contacted via telephone to receive more information about participation and determine eligibility. Individuals were eligible to participate if they were: <15 weeks of gestation; fluent in English; 18–40 years old; not carrying multiples; willing to provide a fasting blood sample and five urine samples throughout pregnancy; did not have a child already participating in IKIDS; resided within a 30-minute drive of the University of Illinois campus; their pregnancy had not been classified as high-risk by their doctor for a reason other than advanced maternal age; and they planned to remain in the area until the child’s first birthday. Those who chose to participate were enrolled at 8–14 weeks of gestation and provided written informed consent. Demographics, pregnancy and health history, pregnancy symptoms, medication use, and lifestyle factors were obtained by interview shortly after enrollment and updated throughout the pregnancy. Written informed consent was also obtained at each age that language development data were collected. IKIDS was approved and overseen by the University of Illinois Urbana-Champaign Institutional Review Board.

### Acetaminophen use during pregnancy

At approximately 10–14, 16–18, 22–24, 28–30, and 34–36 weeks of gestation, and within 24 hours of the child’s birth, participants were interviewed about their medication use. At the first interview (10–14 weeks), participants were asked to list all medications they had used beginning at their estimated conception date through the time of the interview as well as their reason for use (indication), and frequency of use. At subsequent interviews, participants were asked to recall the same information for the period between their last interview and the current one. From these data, medications containing acetaminophen as an active ingredient were identified. Using reported frequency and dates of use, the number of times participants took acetaminophen during the first, second, and third trimesters was calculated. Cumulative use was the number of times acetaminophen was taken across all three trimesters.

### Language measures

Language data included in these analyses were collected between December 2016 and August 2022. When children reached 26.5–28.5 and 36–38 months of age, caregivers were asked to participate in follow-ups of the study child. Those who agreed were mailed a packet of questionnaires which included the CDI^50^ at 26.5–28.5 months, and the SLAS^51^ at 36–38 months.

The CDI is a parental report form which provides measures of the child’s expressive vocabulary, language complexity, and mean length of the longest three utterances (M3L) standardized by sex and age. To measure expressive vocabulary, caregivers indicate which words their child says from a checklist of 680 words. For language complexity, caregivers are asked to indicate which in each pair of 37 sentences sounds more like how their child talks. Caregivers also provide three examples of their child’s longest sentences which are broken down into morphemes (smallest unit of meaning) to calculate M3L. Raw scores were calculated and normalized to percentile scores based on the child’s age and sex. Analyses were conducted using raw scores in generalized linear regression models and percentile scores categorized as above or ≤25th percentile in logistic regression models to calculate odds ratios (ORs).

The SLAS is a short questionnaire with Likert scale questions which evaluate five areas of language use: assertiveness, responsiveness, semantics, syntax, and articulation.^51,53,54^ It has been found to be reliable and correlated with other language development measures, including the CDI. In the initial study evaluating the SLAS, five items pertaining to comprehension, cultural awareness, and speaking too loudly or softly were found to be unreliable. Therefore, subsequent studies have excluded these items.^51,53,54^ Talkativeness is based on one question; thus, most studies have also excluded it.^53,54^ Because each of the scores for the five scales consist of only two or three questions, only the total score, calculated using the 13 items with high inter-rater reliability from these scales, was used in this study.

### Covariates

Acetaminophen formulation and indication were collected only for those who reported taking acetaminophen, thus were not included in models. The following sociodemographic factors were considered for inclusion as covariates in multivariable and logistic regression models based on a priori knowledge and using directed acyclic graphs (Supplementary Fig. S1): parental education, maternal age, parity, household income, tobacco smoking and alcohol use during the first trimester, whether the participant’s native language was English, and child age at assessment and number of older siblings. Additionally, mean perceived stress score (PSS) and Edinburgh Postnatal Depression Scale (EPDS) scores averaged from the following life stages: prenatal, infancy, early childhood, and individual scores at the time of each assessment, were considered. Maternal verbal IQ and birth weight were not considered due to the high amount of missingness (Table 1). Correlations of potential covariates with both exposure and outcome variables were explored, and covariates were included in models when they were correlated with exposure and at least one outcome. In all models using continuous outcomes, child sex was included as a potential modifier. Mean PSS and EPDS scores during pregnancy were included for both the CDI and SLAS. Child age at the time of assessment and maternal parity and education were also included in the CDI models.

**Table 1:** Parental characteristics for all IKIDS participants with an infant enrolled and had exposure data available (*n* = 532), those who provided language data at 26.5–28.5 months (CDI; *n* = 298), and those who provided language data at 36–38 months (SLAS; *n* = 254) by whether they reported taking acetaminophen during pregnancy or not.

Participant Characteristics	Participants with an infant enrolled in study & exposure data available		Participants who completed the CDI when their child was 26.5–28.5 months		Participants who completed the SLAS when their child was 36–38 months	
	Participant did not take acetaminophen during pregnancy	Participant took acetaminophen during pregnancy	Participant did not take acetaminophen during pregnancy	Participant took acetaminophen during pregnancy	Participant did not take acetaminophen during pregnancy	Participant took acetaminophen during pregnancy
	(*n* = 155)	(*n* = 377)	(*n* = 90)	(*n* = 208)	(*n* = 80)	(*n* = 174)
	*N* (%)	*N* (%)	*N* (%)	*N* (%)	*N* (%)	*N* (%)
Maternal race & ethnicity						
White, Non-Hispanic	121 (78.1)	324 (85.9)	71 (78.9)	182 (87.5)	67 (83.8)	156 (89.6)
Other	34 (21.9)	51 (13.5)	19 (21.1)	26 (12.5)	13 (16.2)	17 (9.8)
Unknown/Missing	0 (0.0)	2 (0.5)	0 (0.0)	0 (0.0)	0 (0.0)	1 (0.6)
Other parent’s race & ethnicity						
White, Non-Hispanic	118 (76.1)	318 (84.4)	72 (80.0)	185 (88.9)	65 (81.2)	155 (89.1)
Other	37 (23.9)	57 (15.1)	18 (20.0)	23 (11.1)	15 (18.8)	18 (10.3)
Unknown/Missing	0 (0.0)	2 (0.5)	0 (0.0)	0 (0.0)	0 (0.0)	1 (0.6)
Maternal marital status						
Married/Living as married	143 (92.3)	351 (93.1)	84 (93.3)	198 (95.2)	77 (96.2)	166 (95.4)
Separated/Divorced/ Widowed/Single	12 (7.7)	26 (6.9)	6 (6.7)	10 (4.8)	3 (3.8)	8 (4.6)
Maternal education						
< Bachelor’s degree	25 (16.1)	78 (20.7)	7 (7.8)	27 (13.0)	4 (5.0)	22 (12.6)
≥ Bachelor’s degree	130 (83.9)	299 (79.3)	83 (92.2)	181 (87.0)	76 (95.0)	152 (87.4)
Other parent’s education						
< Bachelor’s degree	41 (26.5)	116 (30.8)	16 (17.8)	45 (21.6)	14 (17.5)	41 (23.6)
≥ Bachelor’s degree	113 (72.9)	259 (68.7)	74 (82.2)	163 (78.4)	66 (82.5)	133 (76.4)
Unknown/Missing	1 (0.7)	2 (0.5)				
Maternal parity						
0	75 (48.4)	198 (52.5)	47 (52.2)	119 (57.2)	41 (51.2)	94 (54.0)
≥1	80 (51.6)	178 (47.2)	43 (47.8)	89 (42.8)	39 (48.8)	79 (45.4)
Missing	0 (0.0)	1 (0.3)	0 (0.0)	0 (0.0)	0 (0.0)	1 (0.6)
Annual household income						
$0–$49,999	33 (21.3)	70 (18.6)	17 (18.9)	29 (13.9)	11 (13.8)	25 (14.4)
$50,000–$99,999	72 (46.4)	181 (48.0)	41 (45.5)	96 (46.2)	37 (46.2)	85 (48.8)
≥$100,000	48 (31.0)	124 (32.9)	32 (35.6)	83 (39.9)	31 (38.8)	64 (36.8)
Unknown/Missing	2 (1.3)	2 (0.5)	0 (0.0)	0 (0.0)	1 (1.2)	0 (0.0)
Maternal health insurance status						
Insured	153 (98.7)	376 (99.7)	90 (100.0)	208 (100.0)	80 (100.0)	174 (100.0)
Uninsured	2 (1.3)	1 (0.3)	0 (0.0)	0 (0.0)	0 (0.0)	0 (0.0)
Maternal tobacco smoking during 1st trimester						
Yes	7 (4.5)	18 (4.8)	3 (3.3)	4 (1.9)	3 (3.8)	8 (4.6)
No	139 (89.7)	333 (88.3)	84 (93.3)	191 (91.8)	72 (90.0)	151 (86.8)
Unknown/Missing	9 (5.8)	26 (6.9)	3 (3.3)	13 (6.3)	5 (6.2)	15 (8.6)
Maternal alcohol during 1st trimester consumption						
Yes	116 (74.8)	278 (73.7)	68 (75.6)	153 (73.6)	59 (73.8)	130 (74.7)
No	39 (25.2)	98 (26.0)	22 (24.4)	55 (26.4)	21 (26.2)	44 (25.3)
Unknown/Missing	0 (0.0)	1 (0.3)	0 (0.0)	0 (0.0)	0 (0.0)	0 (0.0)
Gestational weeks 0–4						
None	96 (61.9)	222 (58.9)	55 (61.1)	121 (58.2)	49 (61.3)	98 (56.3)
<4 drinks/week	46 (29.7)	124 (32.9)	27 (30.0)	72 (34.6)	24 (30.0)	59 (33.9)
4–10 drinks/week	8 (5.2)	25 (6.6)	7 (7.8)	12 (5.8)	9 (11.3)	14 (8.0)
11–21 drinks/week	1 (0.7)	5 (1.3)	1 (1.1)	3 (1.4)	1 (1.3)	3 (1.7)
Unknown/Missing	0 (0.0)	1 (0.3)	0 (0.0)	0 (0.0)	0 (0.0)	0 (0.0)
Gestational weeks 5–8						
None	151 (97.4)	364 (96.6)	87 (96.7)	200 (96.2)	77 (96.3)	166 (95.4)
<4 drinks/week	3 (1.9)	6 (1.6)	2 (2.2)	5 (2.4)	2 (2.5)	3 (1.7)
Unknown/Missing	1 (0.6)	7 (1.9)	1 (1.1)	3 (1.4)	1 (1.3)	5 (2.9)
Gestational weeks 9–14						
None	152 (98.1)	370 (98.1)	88 (97.8)	204 (98.1)	79 (98.8)	171 (98.3)
<4 drinks/week	3 (1.9)	3 (0.8)	2 (2.2)	2 (1.0)	1 (1.3)	1 (0.6)
Unknown/Missing	0 (0.0)	4 (1.1)	0 (0.0)	2 (1.0)	0 (0.0)	2 (1.1)
Maternal non-native English speaker						
Yes	17 (11.0)	10 (2.7)	11 (12.2)	9 (4.3)	8 (10.0)	8 (4.6)
No	138 (89.0)	367 (97.3)	79 (87.8)	199 (95.7)	72 (90.0)	166 (95.4)
Maternal age (years) at baseline	30.82 (4.06)	30.16 (4.13)	31.21 (3.99)	30.55 (3.77)	30.99 (3.79)	30.55 (3.68)
Maternal verbal IQ (PPVT^a^ standardized score)	107.66 (12.92)^d^	107.96 (10.64)^e^	108.26 (12.99)^g^	108.21 (10.19)^h^	109.61 (11.30)^i^	109.24 (10.59)^j^
Mean maternal stress (PSS-10^b^) score during pregnancy	10.61 (5.64)	11.33 (5.69)	10.26 (5.46)	10.82 (5.29)	9.82 (5.18)	10.63 (5.47)
Mean maternal depression (EPDS^c^) score during pregnancy	3.94 (3.05)	4.31 (3.38)	3.89 (2.83)	3.96 (3.15)	3.62 (2.76)	3.96 (3.17)

^a^PPVT-IV: Peabody Picture Vocabulary Test - Fourth Edition.

^b^PSS-10: Perceived Stress Scale.

^c^EPDS: Edinburgh Postnatal Depression Scale

^d^PPVT unable to be collected due to missed visit or COVID-19 *n* = 121.

^e^PPVT unable to be collected due to missed visit or COVID-19 *n* = 47.

^f^PPVT unable to be collected due to missed visit or COVID-19 *n* = 2.

^g^PPVT unable to be collected due to missed visit or COVID-19 *n* = 18.

^h^PPVT unable to be collected due to missed visit or COVID-19 *n* = 38.

^i^PPVT unable to be collected due to missed visit or COVID-19 *n* = 13.

^j^PPVT unable to be collected due to missed visit or COVID-19 *n* = 22.

### Statistical approach

Multivariable generalized linear regression models were used to evaluate the relationship between the number times acetaminophen was taken during each trimester and throughout pregnancy, and each continuous outcome at 26.5–28.5 months (vocabulary size, M3L, and complexity) and 36–38 months (total SLAS score). Because language development is sexually dimorphic, with females developing language earlier and faster than males,^55^ sex-by-exposure interactions were also examined, and when the interaction *p*-value was <0.10, results were also stratified by child sex.

Additionally, the raw CDI scores were converted to percentile scores and categorized as ≤25th or >25th percentile for complementary analyses using multivariable logistic regression. This analysis allowed for characterization of the odds of a child having scores on the low end of the normal distribution (≤25th percentile) for their sex and age with increasing prenatal acetaminophen exposure. Child sex and age were not included in these models because they are accounted for when raw scores are converted to percentile scores; however, results were stratified by child sex.

Sensitivity analyses were used to evaluate the potential impact of several other variables on the associations. These included: maternal alcohol use (Supplementary Table S1), smoking, whether the mother’s native language was English, marital status, postnatal PSS and EPDS scores (average scores during infancy for the CDI, overall average scores for the SLAS, and scores collected at the same time the outcomes were collected), and the other parent’s education. Other sensitivity analyses were conducted excluding participants for various reasons, including: potential leverage points (Cook’s D > 0.04) in multivariable linear regression; observations with high leverage (extreme values on a predictor variable), defined as greater than 0.3, in logistic regression models; children outside of the defined age range at the time the CDI was collected (*n* = 41); and participants who reported taking psychotropics^56^ (CDI *n* = 37; SLAS *n* = 31) or other analgesics^3^ (*n* = 49). An additional sensitivity analysis was performed including only children who had data available at both ages (*n* = 212). All statistical analyses were conducted using SAS software, Version 9.4 of the SAS System for Windows (Copyright © 2013, SAS Institute Inc., Cary, NC).

## Results

### Participation and demographics

**Fig. 1:**
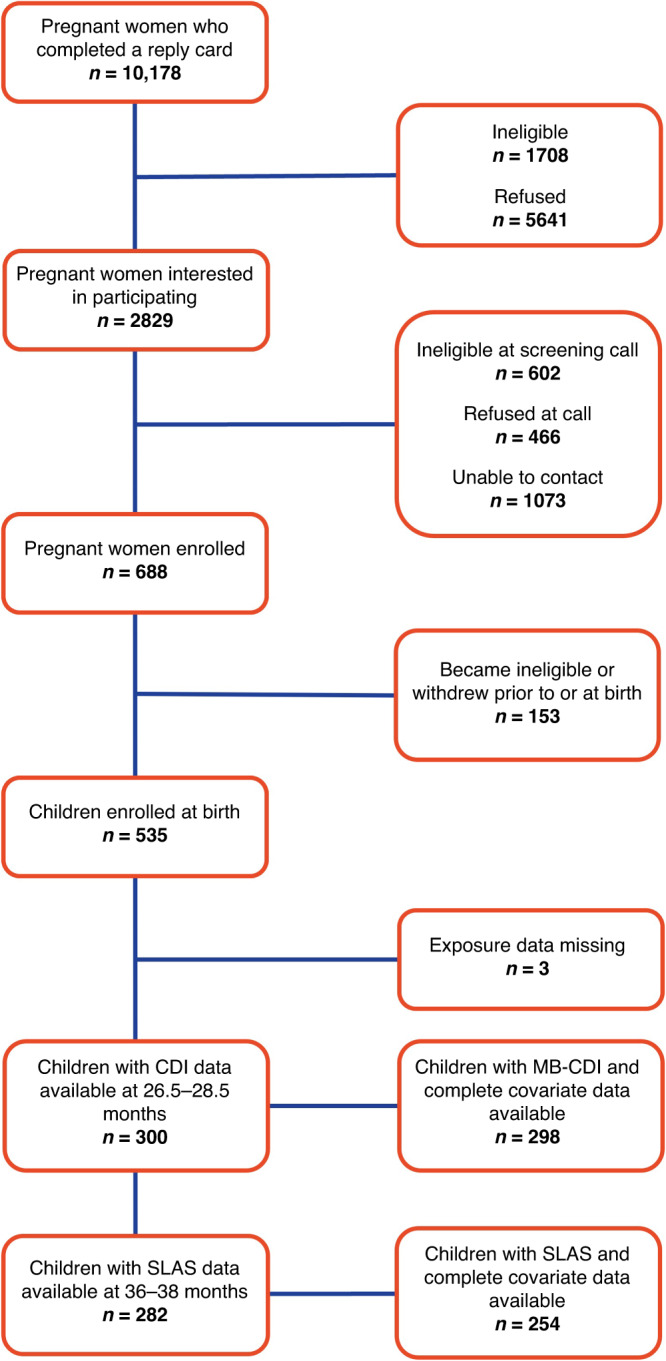
Participant recruitment and inclusion in present analyses. Flowchart of recruitment and retention to IKIDS study visits at 26.5–28.5 and 36–38 months as of January 2023.

By January 2023, 688 pregnant women had been enrolled in the IKIDS cohort, and 153 withdrew or became ineligible before or at the time of birth, resulting in 535 infants born and enrolled in the study (Fig. 1). Three children did not have exposure data available. Demographic information for the 532 participants with an infant enrolled in IKIDS and exposure data available was largely similar to those who completed the CDI at 26.5–28.5 months (*n* = 298) or the SLAS at 36–38 months (*n* = 254), although more were white, non-Hispanic, had attained at least a bachelor’s degree, gave birth vaginally, and had lower mean PSS and EPDS scores during pregnancy in each subsample (Tables 1–2). Participants who reported taking acetaminophen during pregnancy generally did not differ from those who did not (Supplementary Table S2). However, more participants who took acetaminophen were white, non-Hispanic and spoke English as their native language compared to participants who did not take acetaminophen.

**Table 2:** Child demographics for all children enrolled in IKIDS with exposure data available (*n* = 532), those who provided language data at 26.5–28.5 months (CDI; *n* = 254), and those who provided language data at 36–38 months (SLAS; *n* = 254) by whether they reported taking acetaminophen during pregnancy or not.

Child Characteristics	Participants with an infant enrolled in study & exposure data available			Participants who completed the CDI when their child was 26.5–28.5 months			Participants who completed the SLAS when their child was 36–38 months	
	Participant did not take acetaminophen during pregnancy	Participant took acetaminophen during pregnancy	Participant did not take acetaminophen during pregnancy		Participant took acetaminophen during pregnancy	Participant did not take acetaminophen during pregnancy		Participant took acetaminophen during pregnancy
	(*n* = 155)	(*n* = 377)	(*n* = 90)		(*n* = 208)	(*n* = 80)		(*n* = 174)
	*N* (%)	*N* (%)	*N* (%)		*N* (%)	*N* (%)		*N* (%)
Child sex								
Male	76 (49.0)	181 (48.0)	41 (45.6)		110 (52.9)	41 (51.3)		82 (47.1)
Female	79 (51.0)	196 (52.0)	49 (54.4)		98 (47.1)	39 (48.7)		92 (52.9)
Child race & ethnicity								
White, Non-Hispanic	107 (69.0)	296 (78.5)	65 (72.2)		171 (82.2)	61 (76.3)		146 (83.9)
Other	48 (31.0)	79 (21.0)	25 (27.8)		37 (17.8)	19 (23.7)		27 (15.5)
Unknown/Missing	0 (0.0)	2 (0.5)	0 (0.0)		0 (0.0)	0 (0.0)		1 (0.6)
Delivery type								
Vaginal	106 (68.4)	259 (68.7)	64 (71.1)		151 (75.6)	60 (75.0)		122 (70.1)
Cesarean section	37 (23.9)	102 (27.1)	20 (22.2)		53 (25.5)	15 (18.8)		47 (27.0)
Unknown/Missing	12 (7.7)	16 (4.2)	6 (6.7)		4 (1.9)	5 (6.2)		5 (2.9)
	Mean (SD)	Mean (SD)	Mean (SD)		Mean (SD)	Mean (SD)		Mean (SD)
Child gestational age at birth (weeks)	39.37 (1.57)	39.27 (1.45)	39.54 (1.32)		39.32 (1.56)	39.58 (1.33)		39.32 (1.59)
Child weight at birth (kg)	3.46 (0.45)^a^	3.49 (0.42)^b^	3.49 (0.44)^d^		3.48 (0.43)^e^	3.54 (0.47)^f^		3.47 (0.41)^q^

^a^Child weight at birth missing for *n* = 64.

^b^Child weight at birth missing for *n* = 27.

^c^Child weight at birth missing for *n* = 1.

^d^Child weight at birth missing for *n* = 19.

^e^Child weight at birth missing for *n* = 31.

^f^Child weight at birth missing for *n* = 16.

^g^Child weight at birth missing for *n* = 24.

### Acetaminophen use during pregnancy

In IKIDS, 70.9% of participants used a medication containing acetaminophen at least once during pregnancy which is higher than reported in previous studies.^1,4–6^ Few participants took an analgesic other than acetaminophen during pregnancy (Supplementary Table S3). During the first trimester, 58.6% reported use, and fewer participants reported use in subsequent trimesters. Of the participants reporting any use of medications containing acetaminophen, most reported using acetaminophen itself rather than other medications containing acetaminophen, and most took acetaminophen for pain (Table 3). There was no difference in acetaminophen use between the subsets with CDI or SLAS data available and the full sample of children enrolled in IKIDS during the first, second, or third trimesters. Each subset took more acetaminophen throughout pregnancy than the full sample (*p* = 0.01; Supplementary Fig. S2), although the only significant difference was between the full cohort and the subset with SLAS data available (*p* = 0.007; Supplementary Table S4).

**Table 3:** Maternal report of use of medications containing acetaminophen during pregnancy in the IKIDS cohort.

	Participants with an infant enrolled in study & exposure data available				Participants who completed the CDI when their child was 26.5-28.5 months			
	(*n* = 532)				(*n* = 298)			
	1st Trimester	2nd Trimester	3rd Trimester	Entire Pregnancy	1st Trimester	2nd Trimester	3rd Trimester	Entire Pregnancy
	*N* (%)	*N* (%)	*N* (%)	*N* (%)	*N* (%)	*N* (%)	*N* (%)	*N* (%)
None	220 (41.4)	249 (46.8)	338 (63.5)	155 (29.1)	129 (43.3)	148 (49.7)	197 (66.1)	90 (30.2)
Any	312 (58.6)	283 (53.2)	194 (36.5)	377 (70.9)	169 (56.7)	150 (50.3)	101 (33.9)	208 (69.8)
Mean (SD) number of times taken	17.9 (39.7)	22.0 (44.1)	15.1 (25.6)	39.1 (75.1)	14.5 (26.8)	20.1 (44.9)	13.2 (21.6)	32.7 (66.0)
Median (IQR) number of times taken	5.0 (14.0)	7.5 (15.0)	5.0 (12.0)	14.0 (31.8)	6.0 (13.5)	7.0 (13.8)	4.8 (11.3)	12.4 (29.6)
Range number of times taken	0.5–314.0	0.5–350.0	0.5–153.0	1.0–641.0	1.0–248.5	0.5–339.0	0.5–119.5	1.0–550.5
Formulation								
Paracetamol alone	258 (82.7)	229 (80.9)	158 (81.4)	287 (76.1)	140 (82.8)	121 (80.7)	83 (82.2)	158 (76.0)
Paracetamol in combination	25 (8.0)	19 (6.7)	17 (8.8)	26 (6.9)	16 (9.5)	15 (10.0)	10 (9.9)	19 (9.1)
with other active ingredients								
Multiple formulations	29 (9.3)	35 (12.4)	19 (9.8)	64 (17.0)	13 (7.7)	14 (9.3)	8 (7.9)	31 (14.9)
Indication								
Pain	214 (68.6)	202 (71.4)	134 (69.1)	235 (62.3)	110 (65.1)	102 (68.0)	68 (67.3)	118 (56.7)
Other	39 (12.5)	31 (11.0)	26 (13.4)	54 (14.3)	23 (13.6)	20 (13.3)	14 (13.9)	36 (17.3)
Multiple reasons given	27 (8.7)	28 (9.9)	19 (9.8)	61 (16.2)	16 (9.5)	12 (8.0)	10 (9.9)	36 (17.3)
Missing	32 (10.3)	22 (7.8)	15 (7.7)	27 (7.2)	20 (11.8)	16 (10.7)	9 (8.9)	18 (8.7)

	Participants who completed the SLAS when their child was 36–38 months			
	(*n* = 254)			
	1st Trimester	2nd Trimester	3rd Trimester	Entire Pregnancy
	*N* (%)	*N* (%)	*N* (%)	*N* (%)
None	111 (43.7)	123 (48.4)	169 (66.5)	80 (31.5)
Any	143 (56.3)	131 (51.6)	85 (33.5)	174 (68.5)
Mean (SD) number of times taken	13.2 (27.1)	21.9 (47.6)	14.7 (24.2)	34.5 (70.2)
Median (IQR) number of times taken	4.5 (11.0)	8.0 (14.5)	5.0 (12.0)	13.0 (29.0)
Range number of times taken	1.0–248.5	0.5–339.0	0.5–119.5	1.0–550.5
Formulation				
Acetaminophen alone	118 (82.5)	105 (80.2)	69 (81.2)	133 (76.4)
Acetaminophen in combination	15 (10.5)	13 (9.9)	9 (10.6)	16 (9.2)
with other active ingredients				
Multiple formulations	10 (7.0)	13 (9.9)	7 (8.2)	25 (14.4)
Indication				
Pain	92 (64.3)	86 (65.6)	56 (65.9)	96 (55.2)
Other	22 (15.4)	18 (13.7)	12 (14.1)	31 (17.8)
Multiple reasons given	10 (7.0)	12 (9.2)	7 (8.2)	29 (16.7)
Missing	19 (13.3)	15 (11.5)	10 (11.8)	18 (10.3)

*SD* standard deviation, *IQR* interquartile range.

### Language development

As of January 2023, 298 children had CDI outcome data (147 males, 151 females). Most children were 26.5–28.5 months of age with an average age of 27.42 (±1.08) months at the time of assessment. Data for 41 children were collected outside this age range. Not all participants completed all three outcome measures. As expected, females were more advanced in language development than males at this age (vocabulary *p* < 0.0001, M3L *p* = 0.0003, complexity *p* = 0.003, Supplementary Fig. S3, Table S5). Most children who had SLAS data available (*n* = 254; 133 females, 121 males) were 36–38 months at the time of assessment, with an average age of 37.52 (±1.00) months and data for 30 children collected outside this assessment window. Females had higher scores than males, although the sex difference was not as pronounced at this age (*p* = 0.07, Supplementary Fig. S4, Table S5).

### Associations with CDI outcomes at 26.5–28.5 months

**Fig. 2:**
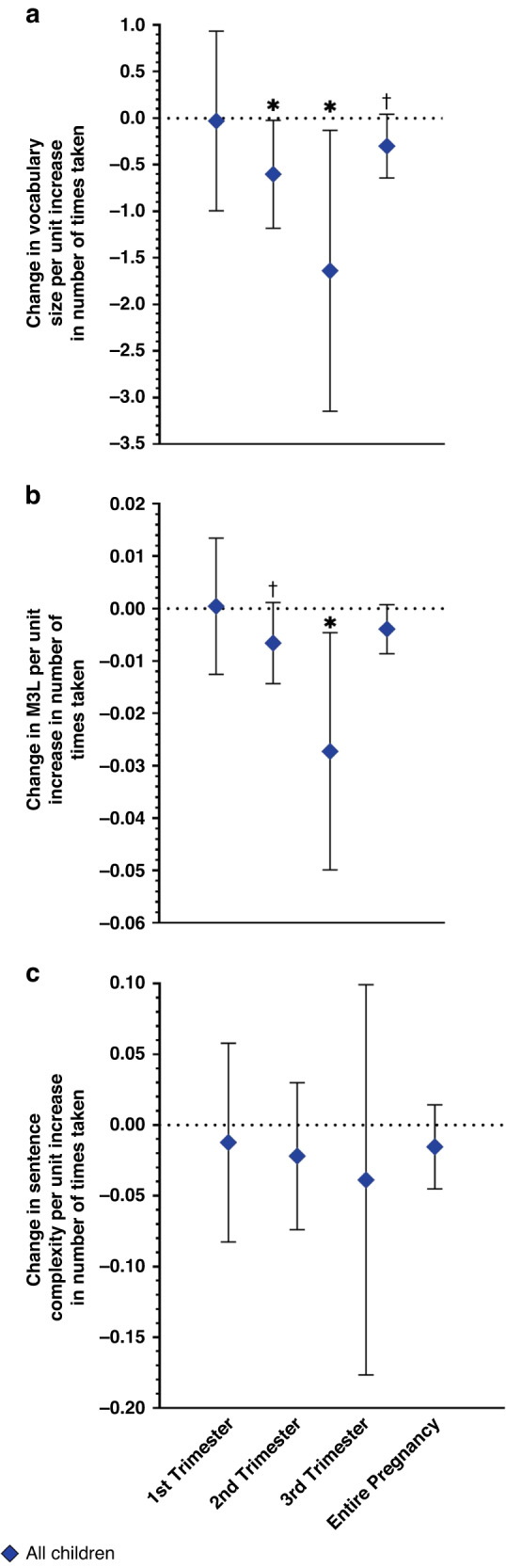
Associations of prenatal acetaminophen exposure and language development outcomes on the CDI^a^. Associations of prenatal acetaminophen exposure and (**a**) change in vocabulary count (number of words produced), (**b**) mean length of three longest utterances (number of morphemes produced, M3L), and (**c**) complexity (number of more complex sentences produced) measured using the CDI at 26.5–28.5 months. More acetaminophen use during the second and third trimesters, and throughout pregnancy, was associated with a decrease in vocabulary size. Increased acetaminophen use in the second and third trimesters was associated with a decrease in M3L. Prenatal acetaminophen exposure was not related to complexity scores. ^a^Models were adjusted for child sex, age at assessment, maternal parity (nulliparous vs. ≥1), maternal education (<bachelor’s degree vs. ≥bachelor’s degree), and mean stress and depression scores during pregnancy. ^†^*p* < 0.10, *p < 0.05

Generally, an increase in the number of times acetaminophen was taken during pregnancy was associated with lower vocabulary size, M3L, and complexity at 26.5–28.5 months of age (Fig. 2, Table 4, Supplementary Figs. S5–6). Associations of acetaminophen use during pregnancy with these outcomes did not differ by child sex. Per unit increase in acetaminophen use (i.e., report of number of times taking acetaminophen) during the second and third trimester, children showed a decrease in vocabulary size of 0.58 (95% CI: −1.13, −0.04) and of 1.83 words (95% CI: −3.13, −0.54), respectively. There was also an association of the number of times acetaminophen was taken across the entire pregnancy with smaller vocabularies (β = −0.35 words, 95% CI: −0.67, 0.03). Similar findings were observed for associations between acetaminophen use and M3L. An increase in acetaminophen use during the second or third trimester was associated with a small decrease in M3L (β = −0.007 morphemes, 95% CI: −0.01, 0.001; β = −0.02 morphemes, 95% CI: −0.04, 0.003). There were no associations of acetaminophen use during the first trimester with any outcomes, and complexity scores were not related to acetaminophen use during pregnancy. Associations were largely unchanged in sensitivity analyses (Supplementary Table S6).

**Table 4:** Multivariable linear regression analyses of the relation (β estimate and 95% confidence interval) of prenatal acetaminophen exposure with language outcomes on the MB-CDI at 2 years.

Outcome			Stratified by Sex	
Unadjusted^a^	Exposure	Main Effect	Females	Males
Vocabulary	1st Trimester	−0.44 (−1.31, 0.43)	−0.66 (−2.18, 0.85)	−0.33 (−1.39, 0.73)
	2nd Trimester	*−0.69 (−1.24, −0.14)	−0.83 (−1.57, −0.09)	−0.51 (−1.34, 0.31)
	3rd Trimester	**−1.95 (−3.26, −0.65)	−1.89 (−3.59, −0.19)	−2.05 (−4.09, 0.003)
	Entire Pregnancy	*−0.41 (−0.74, −0.09)	−0.50 (−0.94, −0.05)	−0.33 (−0.79, 0.13)
Mean Length of Utterance	1st Trimester	−0.005 (−0.02, 0.01)	−0.004 (−0.03, 0.02)	−0.01 (−0.02, 0.01)
	2nd Trimester	^†^−0.007 (−0.01, 0.001)	−0.01 (−0.02, 0.002)	−0.005 (−0.02, 0.01)
	3rd Trimester	*−0.02 (−0.04, −0.0004)	−0.02 (−0.05, 0.001)	−0.02 (−0.05, 0.02)
	Entire Pregnancy	^†^−0.004 (−0.01, 0.001)	−0.005 (−0.01, 0.001)	−0.003 (−0.01, 0.004)
Complexity	1st Trimester	−0.04 (−0.11, 0.02)	−0.05 (−0.16, 0.07)	−0.04 (−0.12, 0.03)
	2nd Trimester	−0.02 (−0.07, 0.02)	−0.03 (−0.11, 0.06)	−0.02 (−0.08, 0.04)
	3rd Trimester	−0.04 (−0.15, 0.07)	−0.01 (−0.16, 0.14)	−0.08 (−0.26, 0.09)
	Entire Pregnancy	−0.02 (−0.04, 0.01)	−0.02 (−0.06, 0.03)	−0.02 (−0.05, 0.01)
Adjusted^b^				
Vocabulary	1st Trimester	−0.28 (−1.14, 0.58)	−0.48 (−1.97, 1.02)	−0.18 (−1.23, 0.87)
	2nd Trimester	*−0.58 (−1.13, −0.04)	−0.78 (−1.52, −0.05)	−0.33 (−1.16, 0.50)
	3rd Trimester	**−1.83 (−3.13, −0.54)	−1.78 (−3.46, −0.11)	−1.91 (−3.93, 0.11)
	Entire Pregnancy	*−0.35 (−0.67, −0.03)	−0.45 (−0.90, −0.01)	−0.24 (−0.70, 0.22)
Mean Length of Utterance	1st Trimester	−0.002 (−0.01, 0.01)	−0.002 (−0.02, 0.02)	−0.003 (−0.02, 0.01)
	2nd Trimester	^†^−0.01 (−0.01, 0.001)	−0.01 (−0.02, 0.001)	−0.003 (−0.02, 0.01)
	3rd Trimester	*−0.02 (−0.04, −0.003)	−0.02 (−0.05, −0.001)	−0.02 (−0.06, 0.01)
	Entire Pregnancy	−0.004 (−0.01, 0.001)	−0.01 (−0.01, 0.001)	−0.002 (−0.01, 0.005)
Complexity	1st Trimester	−0.03 (−0.10, 0.03)	−0.04 (−0.15, 0.07)	−0.03 (−0.10, 0.05)
	2nd Trimester	−0.02 (−0.07, 0.03)	−0.02 (−0.10, 0.06)	−0.01 (−0.07, 0.05)
	3rd Trimester	−0.04 (−0.15, 0.08)	−0.0003 (−0.15, 0.15)	−0.08 (−0.26, 0.09)
	Entire Pregnancy	−0.01 (−0.04, 0.01)	−0.01 (−0.06, 0.03)	−0.01 (−0.05, 0.02)

^a^Unadjusted models were adjusted for child sex and age.

^b^All models were adjusted for child sex and age at assessment, maternal parity, maternal education, mean perceived stress during pregnancy, and mean depression during pregnancy.

^†^*p* < 0.10; *p < 0.05; **p < 0.01.

**Fig. 3:**
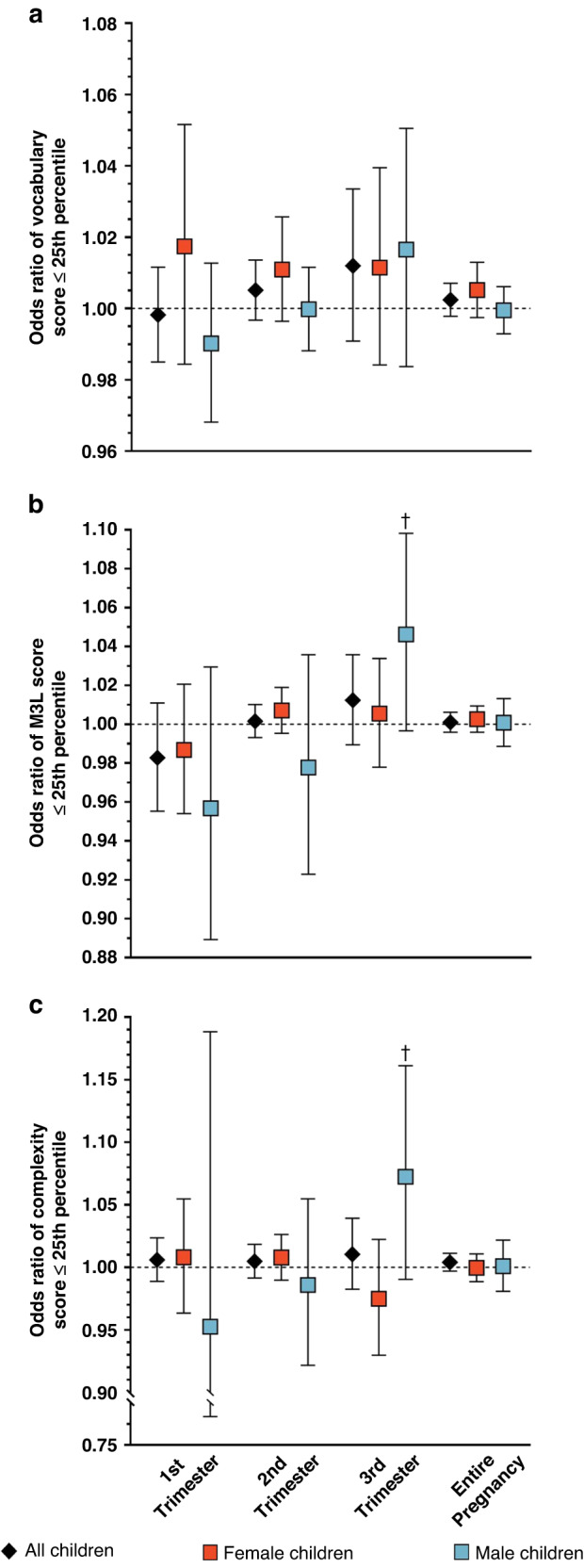
Odds of CDI scores ≤25th percentile in relation to prenatal acetaminophen exposure^a^. Odds ratios and 95% confidence intervals for the relationship of prenatal acetaminophen exposure and (**a**) vocabulary size, (**b**) mean length of three longest utterances (M3L), and (**c**) complexity ≤25th percentile measured using the CDI at 26.5–28.5 months. The number of times acetaminophen was taken was not related to the odds of children having vocabulary scores ≤25th percentile. However, the number of times acetaminophen was taken during the third trimester was associated with slightly increased odds of male children having M3L and complexity scores ≤25th percentile. ^a^Models were adjusted for maternal parity (nulliparous vs. ≥1), maternal education (<bachelor’s degree vs. ≥bachelor’s degree), and mean stress and depression scores during pregnancy. ^†^*p* < 0.10.

In this cohort, 80 children (27%) had vocabulary scores ≤25th percentile, 44 (15%) had M3L scores ≤25th percentile, and 32 (11%) had complexity scores ≤25th percentile for their sex and age. Results of the logistic regression analyses were more mixed, but most showed that an increase in the number of times acetaminophen was taken during pregnancy was associated with an elevated odds of children having vocabulary, M3L, and complexity scores ≤25th percentile (Fig. 3, Table 5). In stratified analyses, increasing acetaminophen use was associated with moderately elevated odds of both M3L (OR = 1.07, 95% CI: 1.01, 1.09) and complexity (OR = 1.07, 95% CI: 1.01, 1. 13) scores ≤25th percentile specifically in male children. Associations were generally unchanged in sensitivity analyses (Supplementary Table S7).

**Table 5:** Odds ratios (odds, 95% CI) of children with prenatal acetaminophen exposure having a percentile score ≤25th percentile adjusted for child sex and age.

Outcome			Stratified by Sex	
Unadjusted	Exposure	Main Effect	Females	Males
Vocabulary	1st Trimester	1.003 (0.99, 1.01)	^†^1.02 (0.99, 1.04)	0.99 (0.98, 1.01)
	2nd Trimester	1.01 (0.99, 1.01)	1.01 (0.99, 1.02)	1.004 (0.99, 1.01)
	3rd Trimester	^†^1.02 (0.99, 1.03)	1.02 (0.99, 1.04)	1.01 (0.99, 1.04)
	Entire Pregnancy	1.003 (0.99, 1.01)	^†^1.01 (0.99, 1.01)	1.001 (0.99, 1.01)
Mean Length of Utterance	1st Trimester	0.996 (0.98, 1.01)	0.99 (0.97, 1.02)	0.99 (0.96, 1.02)
	2nd Trimester	1.004 (0.99, 1.01)	1.01 (0.99, 1.02)	0.99 (0.98, 1.02)
	3rd Trimester	^†^1.02 (0.99, 1.04)	1.01 (0.99, 1.03)	^†^1.03 (0.99, 1.07)
	Entire Pregnancy	1.002 (0.99, 1.01)	1.003 (0.99, 1.01)	1.001 (0.99, 1.01)
Complexity	1st Trimester	1.003 (0.99, 1.02)	1.01 (0.99, 1.03)	0.93 (0.77, 1.12)
	2nd Trimester	1.001 (0.99, 1.01)	1.01 (0.99, 1.02)	0.99 (0.92, 1.06)
	3rd Trimester	1.02 (0.99, 1.04)	1.003 (0.97, 1.03)	*1.04 (1.01, 1.09)
	Entire Pregnancy	1.002 (0.99, 1.01)	1.004 (0.99, 1.01)	1.00 (0.99, 1.01)
Adjusted^a^				
Vocabulary	1st Trimester	1.002 (0.99, 1.01)	1.02 (0.99, 1.04)	0.99 (0.98, 1.01)
	2nd Trimester	1.01 (0.99, 1.01)	1.01 (0.99, 1.02)	1.003 (0.99, 1.01)
	3rd Trimester	^†^1.02 (0.99, 1.03)	1.02 (0.99, 1.04)	1.01 (0.99, 1.04)
	Entire Pregnancy	1.003 (0.99, 1.01)	1.01 (0.99, 1.01)	1.001 (0.99, 1.01)
Mean Length of Utterance	1st Trimester	0.99 (0.98, 1.01)	0.99 (0.97, 1.02)	0.99 (0.96, 1.03)
	2nd Trimester	1.003 (0.99, 1.01)	1.004 (0.99, 1.01)	0.99 (0.98, 1.02)
	3rd Trimester	^†^1.02 (0.997, 1.04)	1.01 (0.98, 1.03)	*1.05 (1.01, 1.09)
	Entire Pregnancy	1.002 (0.99, 1.01)	1.002 (0.99, 1.01)	1.003 (0.99, 1.01)
Complexity	1st Trimester	1.01 (0.99, 1.02)	1.01 (0.99, 1.03)	0.95 (0.78, 1.15)
	2nd Trimester	1.003 (0.99, 1.02)	1.003 (0.99, 1.02)	0.99 (0.93, 1.05)
	3rd Trimester	1.02 (0.99, 1.04)	1.003 (0.97, 1.04)	*1.07 (1.01, 1.13)
	Entire Pregnancy	1.003 (0.99, 1.01)	1.003 (0.99, 1.01)	1.004 (0.99, 1.02)

^a^All models were adjusted for maternal parity, maternal education, mean perceived stress during pregnancy, and mean depression during pregnancy.

^†^*p* < 0.10; *p < 0.05; **p < 0.01.

### Associations with total SLAS score at 36–38 months

**Fig. 4:**
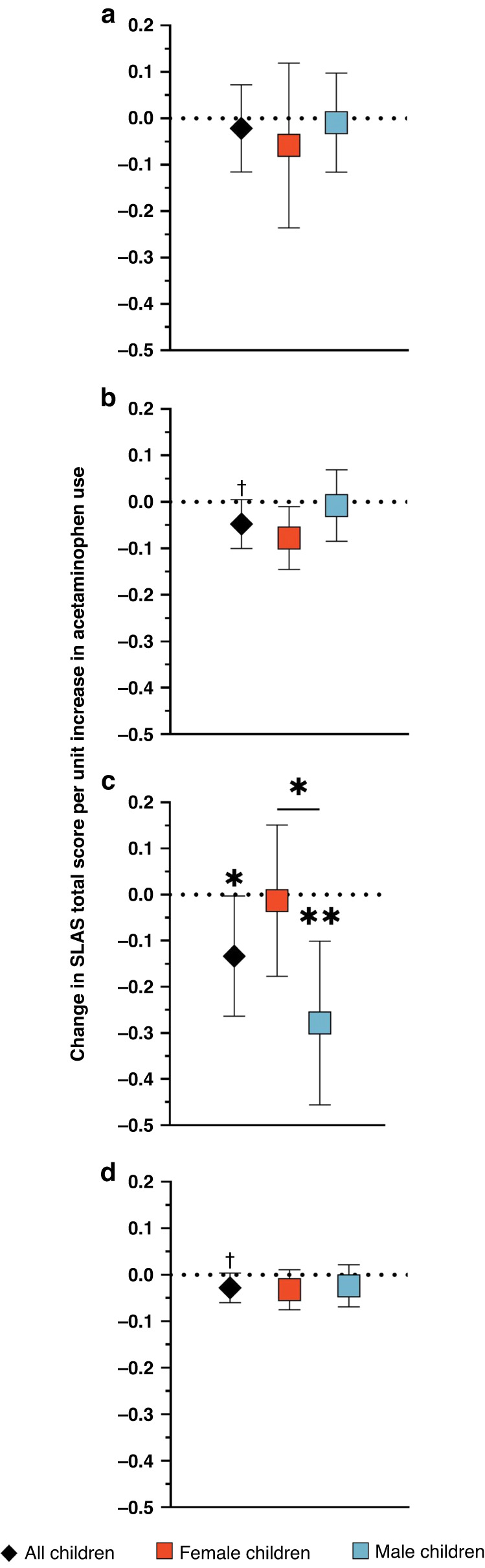
Associations of prenatal acetaminophen exposure and SLAS total score at 36–38 months by trimester of exposure^a^. More acetaminophen use during (**a**) the first trimester was not related to SLAS scores. More frequent use during the (**b**) second and (**c**) third trimesters, and (**d**) throughout pregnancy, was associated with lower SLAS total scores in all children. More acetaminophen use during third trimester was also related to a decrease in total SLAS scores in male children specifically. ^a^Models were adjusted for child sex and mean stress and depression scores during pregnancy. ^†^*p* < 0.10, *p < 0.05, **p < 0.01.

An increase in acetaminophen use during pregnancy was generally associated with lower total SLAS scores at 36–38 months of age, and associations mostly did not differ by child sex (Fig. 4, Table 6, Supplementary Fig. S7). An increase in acetaminophen use during the second (β = −0.04, 95% CI: −0.09, 0.007) and third trimesters (β = −0.15, 95% CI: −0.26, −0.04), as well as across the entire pregnancy (β = −0.03, 95% CI: −0.06, −0.001) was associated with a decrease in SLAS scores. The sex-by-acetaminophen use interaction was *p* < 0.10 only for third trimester exposure, with males having significantly lower SLAS scores (β = −0.27, 95% CI: −0.43, −0.11; Supplementary Fig. S8) compared to females (β = −0.04, 95% CI: −0.20, 0.11). Associations were not substantially altered in sensitivity analyses (Supplementary Table S8).

**Table 6:** Multivariable linear regression analyses of the relation (β estimate and 95% confidence interval) of prenatal acetaminophen exposure with language outcomes on the SLAS at 3 years.

Unadjusted^a^		Stratified by Sex	
Exposure	Main Effect	Females	Males
1st Trimester	^†^−0.07 (−0.16, 0.01)	−0.12 (−0.27, 0.03)	−0.05 (−0.15, 0.06)
2nd Trimester	*−0.06 (−0.11, −0.01)	−0.09 (−0.15, −0.02)	−0.03 (−0.10, 0.05)
3rd Trimester	**−0.18 (−0.29, −0.06)	^‡^−0.08 (−0.24, 0.08)	**−0.29 (−0.45, −0.12)
Entire Pregnancy	**−0.04 (−0.07, −0.01)	−0.05 (−0.09, −0.01)	−0.04 (−0.08, 0.01)
Adjusted^b^			
1st Trimester	−0.04 (−0.13, 0.04)	−0.11 (−0.25, 0.04)	−0.01 (−0.11, 0.09)
2nd Trimester	^†^−0.04 (−0.09, 0.01)	−0.07 (−0.14, −0.01)	−0.002 (−0.08, 0.07)
3rd Trimester	**−0.15 (−0.26, −0.04)	^§^−0.04 (−0.198, 0.11)	**−0.27 (−0.43, −0.11)
Entire Pregnancy	*−0.03 (−0.06, −0.001)	−0.04 (−0.08, 0.001)	−0.02 (−0.07, 0.02)

^a^Unadjusted models were adjusted for child sex and age.

^b^All models were adjusted for child sex, maternal education, mean perceived stress during pregnancy, and mean depression during pregnancy.

Interaction *p*-values: ^‡^*p* < 0.10, ^§^*p* < 0.05.

*p < 0.05; **p < 0.01.

## Discussion

Generally, more acetaminophen use during pregnancy was associated with modest decreases in early childhood language outcomes in this largely white, non-Hispanic, well-educated, and high-income cohort. Sex-specific associations were observed between increased acetaminophen use late in pregnancy and language outcomes in male children. Increased frequency of acetaminophen use during the second and third trimester were associated with slightly smaller vocabularies and shorter M3L when children were 26.5–28.5 months. An increase in the total number of times acetaminophen was taken throughout pregnancy was also related to slightly shorter M3L. Additionally, increased use during the third trimester was related to elevated odds of male children having M3L and complexity scores ≤25th percentile. Finally, increases in the number of times acetaminophen was taken during the second and third trimesters were related to small decreases in total SLAS scores in male children.

These results align with previous research examining the relationship between prenatal acetaminophen exposure and communication or language outcomes. Using samples of the same cohort, two studies found that acetaminophen exposure during pregnancy was associated with poorer communication skills at 3 years.^4,49^ A third observed a similar association earlier in children 18 months of age;^31^ however, all three studies used the ASQ. While there is substantial evidence that the ASQ has high specificity, reliability, and validity,^57–60^ the communication section has lower specificity, sensitivity, and accuracy than the CDI,^61,62^ and does not evaluate language development as extensively or in as much detail as the CDI. Only one previous study observed sex-specific effects, but, unlike this study, they found greater language delays in female children according to a routine nurse evaluation at 30 months.^48^ This difference may be related to the timing of exposure as that study only examined acetaminophen exposure during the first trimester, and the associations with greater acetaminophen use observed here for male children were only found during the third trimester. This suggests that additional research examining the timing of prenatal acetaminophen use in relation to child sex is necessary to better understand the role trimester of use plays in sex-specific associations with poorer language outcomes.

These results also suggest that the second and third trimesters may be windows of neurodevelopment particularly sensitive to disruption of language development by prenatal acetaminophen exposure. Few previous studies have evaluated whether timing of acetaminophen use during pregnancy played a role, but similarly, Brandlistuen et al. (2013)^4^ observed an association of more days of acetaminophen exposure during the third trimester with poorer communication skills in 3-year-old children. Several structures important in auditory pathways in the brain rapidly develop during the second trimester of fetal development, which could be why acetaminophen use during this period was associated with measures of language development.^63–65^ In particular, the tympanic membrane,^66^ middle ear,^67,68^ and much of the inner ear are rapidly developing during the second trimester,^65,69–71^ at which time, fetuses also begin responding to sound.^71–73^ Axons of brainstem auditory neurons also mature during the second trimester,^65,74,75^ followed by myelination, which begins towards the end of the second trimester.^65,76,77^ At the beginning of the third trimester, axonal conduction time along the auditory nerve rapidly develops, at which time hearing onset is reliably observed in healthy fetuses, and conduction time in brainstem pathways begins to mature.^65,74,77^ During the second trimester, the cortex develops from a thin-walled vesicle lined with densely packed immature neurons into a structure more similar to a mature cortex with distinct cortical layers.^65,74^ As cortical neurons continue to develop during the third trimester, the temporal lobe forms into a distinct structure with the primary auditory cortex contained within Heschl’s gyrus and the secondary auditory cortex, which includes Wernicke’s area, in the superior temporal gyrus.^65,78^ All of this lays the groundwork for further development and maturation necessary for language processing and acquisition. Recent evidence suggests that the analgesic effect of acetaminophen occurs via the endocannabinoid system, which plays an important role in multiple aspects of neurodevelopment, including cell differentiation, migration, and synaptogenesis.^79^ Thus, use of acetaminophen during the second or third trimester may interfere with the development of auditory structures and pathways via the endocannabinoid system.

Because of the way the acetaminophen use was ascertained, previous studies have not been able to examine associations by trimester, whereas participants in the current study reported acetaminophen use six times across pregnancy, allowing for evaluation by trimester of use, and a reduced risk of inaccuracies in reporting. There were clear associations of increased frequency of acetaminophen use with poorer language outcomes despite the small sample size of the current study. However, given the importance of acetaminophen as an analgesic, these findings should be interpreted cautiously until they are replicated in a larger and more diverse sample. The changes in language scores were also small with the largest being a decrease in vocabulary size by almost 2 words per number of times acetaminophen was taken during the third trimester. This suggests that if a pregnant person took acetaminophen thirteen times (or approximately once per week) during the third trimester, their child may express approximately 26 fewer words than other children of the same age.^51^ Similarly, the child may produce 0.33 fewer morphemes, and if the child was male, they would have 65% greater odds of having a M3L score ≤25th percentile and a 91% greater odds of having a complexity score ≤25th percentile. For the same scenario, children could have a nearly 2-point decrease in total SLAS scores, or a 3.6-point decrease if the child was male. While the estimates are small, the majority of participants in this cohort are of higher socioeconomic status, and individuals of lower socioeconomic status may show larger deficits.^80,81^ Further, even small effects measured in a sample may have a substantial impact at the population level, particularly when the exposure is common.^82^

While the CDI asks caregivers to evaluate the child’s language objectively, the SLAS asks parents to rate their child’s language skills relative to other children of the same age. Despite differences in the assessment measures used in this study, increased use of acetaminophen during pregnancy was associated with lower scores on both measures. Previous studies have demonstrated that parents are fairly accurate in their assessment of their child’s language abilities,^50,83,84^ and the initial studies introducing the SLAS also found that parental and Speech Language Pathologist ratings of a child’s language abilities using the SLAS were highly correlated with one another.^51^ Additionally, the SLAS has since been selected as a recommended protocol by the PhenX (consensus measures for Phenotypes and eXposures) Toolkit,^85^ a database of measures for a variety of developmental domains put together by expert review panels intended to facilitate research across studies, because it is short, easy to use and interpret, and is both well-established and validated.^86^

### Strengths and limitations

This study has several strengths. First, it utilizes data from an ongoing prospective birth cohort study which continues to follow these children. Second, this study ascertained acetaminophen use multiple times during pregnancy, reducing the risk of memory inaccuracies, and allowing for consideration of acetaminophen use by trimester. Results were also robust to several sensitivity analyses, suggesting these associations may not be driven by extreme values for the number of times acetaminophen was taken. Another strength is the use of multiple measures of language development at different ages. This study is the first to our knowledge to utilize the CDI or SLAS to evaluate associations of acetaminophen use during pregnancy with language development in children. CDI scores at 2 years have been shown to be highly correlated with SLAS scores later in early childhood, and both measures have also been shown to be consistent with children’s performance on other standardized language assessments.^53,54^

This study also has limitations. Many analyses were conducted without correction for multiple comparisons. In epidemiology, it has been argued that the focus should be on looking for trends in results to better-inform future research,^87^ which was the approach taken here. Second, this cohort is relatively homogenous, limiting the generalizability of the findings. As is the case with cohort studies, this study is also subject to loss to follow-up, with participants remaining in the study tending to be white, non-Hispanic, well-educated with a high annual household income, and have lower EPDS and PSS scores. In this analysis, only 56% of participants with an infant enrolled at birth contributed CDI data, and while not all children enrolled had yet reached the 36-38 month assessment, only 48% contributed SLAS data. There is also a risk of dependent error due to the mother reporting both the exposure and, in almost all cases, outcomes. While there were multiple times of self-report of medication use during pregnancy, it is likely these reports were not entirely accurate due to some inaccuracies in memory. Additionally, dosage information was not collected and thus could not be assessed in this study. Unfortunately, whether participants experienced the indications (such as pain) but did not take acetaminophen or another analgesic was not available, and therefore could not be investigated. As such, the indications participants provided for taking acetaminophen cannot be ruled out as potentially contributing to the lower scores observed in this study. To our knowledge, the relationship of pain during pregnancy with neurodevelopment has not yet been investigated, but inflammation is commonly associated with pain as well as infections. Both inflammation and infections during pregnancy have been repeatedly linked to Autism Spectrum Disorder (ASD), a condition in which language is often impaired.^88^ However, one study found that diagnosed infection during pregnancy was not associated with ASD but treatment for infection was, and the authors note that the relationship of prenatal infection, treatment, and ASD could not be disentangled.^89^ Although a few studies have attempted to utilize biomarkers of acetaminophen exposure in maternal urine, maternal plasma, umbilical cord blood, or meconium, these have their own limitations.^6,40,48,90,91^ One promising, yet un-investigated, biomarker is shed teeth in which acetaminophen use during infancy can be measured.^92^ This should be explored as a biomarker of prenatal acetaminophen exposure in the future.^83^ Despite these limitations, the results of this study suggest that the impact of acetaminophen use during pregnancy on child neurodevelopment should be further investigated.

### Importance of findings

Acetaminophen is the most common drug ingredient on the market, and one of very few medications considered safe for use throughout pregnancy.^1^ However, there is a growing body of literature indicating that its use during pregnancy may be related to poorer neurodevelopmental outcomes. As recently concluded in a systematic review, the idea that it is safe for use has been taken for granted for many years without any demonstration of its safety regarding neurodevelopment.^8^ This study provides additional evidence that more acetaminophen use during pregnancy is related to poorer language development^93^ and highlights the need for further investigation of the potential mechanisms through which prenatal acetaminophen exposure may impact neurodevelopment, as well as further investigation using larger and more diverse cohort studies to establish whether there is strong causal evidence for this association.

### Supplementary information

Supplementary Information

Supplementary Table

## Data Availability

The data used in these analyses cannot be shared publicly to ensure the anonymity of participants. Underlying code is available upon request.
