# Supplementary material for: Examining the relationship of acetaminophen use during pregnancy with early language development in children
Source: Pediatr Res. 2023 Dec 11;95(7):1883–96. doi: 10.1038/s41390-023-02924-4 (PMC11164826; doi:10.1038/s41390-023-02924-4)
Supplement: Supplementary file 1 — Supplementary Information [file 41390_2023_2924_MOESM1_ESM.pdf]

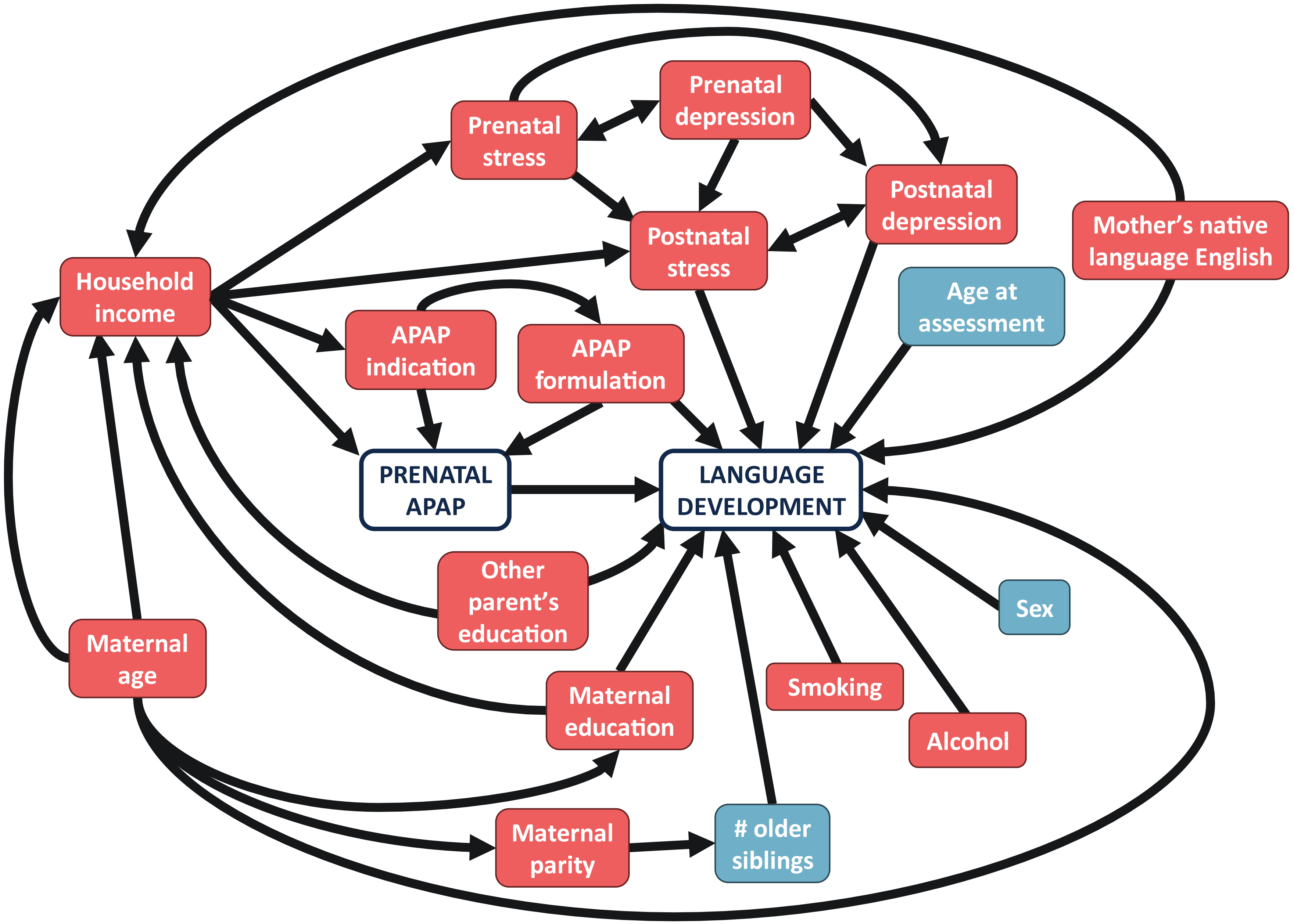

Supplemental Figure S1. Directed acyclic graph utilized in selecting covariates to consider for inclusion in models. APAP is the abbreviation for acetaminophen. For the purpose of simplicity, mean stress and depression across infancy and childhood as well as at each age at which language was assessed are all included in "Postnatal stress" and "Postnatal depression." Blue boxes indicate child-related variables, and coral boxes indicate parent-related variables.

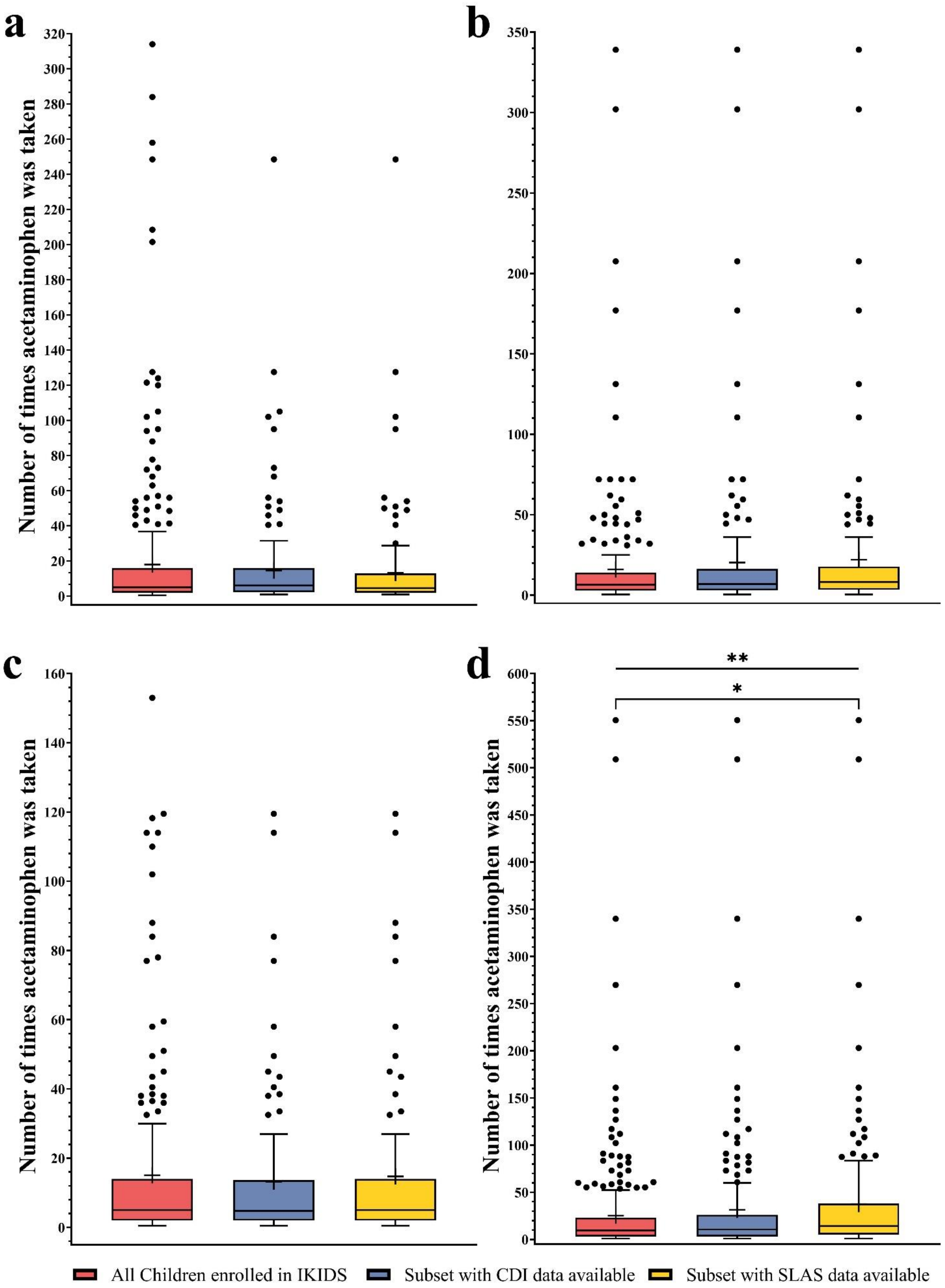

Supplemental Figure S2. Box and whisker plots showing the distribution, including mean (+), median, and outliers, of acetaminophen use during the (a) first trimester, (b) second trimester, (c) third trimester, and (d) entire pregnancy by the full sample of children enrolled in IKIDS at birth, the

subset of children with CDI data available, and the subset with SLAS data available. Kruskal-Wallis Tests indicated there was no difference in use among the three groups (full sample, CDI subset, and SLAS subset) for first trimester use ($p = 0.60$), second trimester use ($p = 0.13$), or third trimester use ($p = 0.79$), but they did differ for use during the entire pregnancy ($p = 0.01$). Wilcoxon-Rank Sum Tests revealed there was a significant difference in use between the full sample and the subset with SLAS data available ($p = 0.007$), but no difference between the full sample and the subset with SLAS data available or the subset with CDI data available and the subset with SLAS data available.

*p < 0.05
**p < 0.01

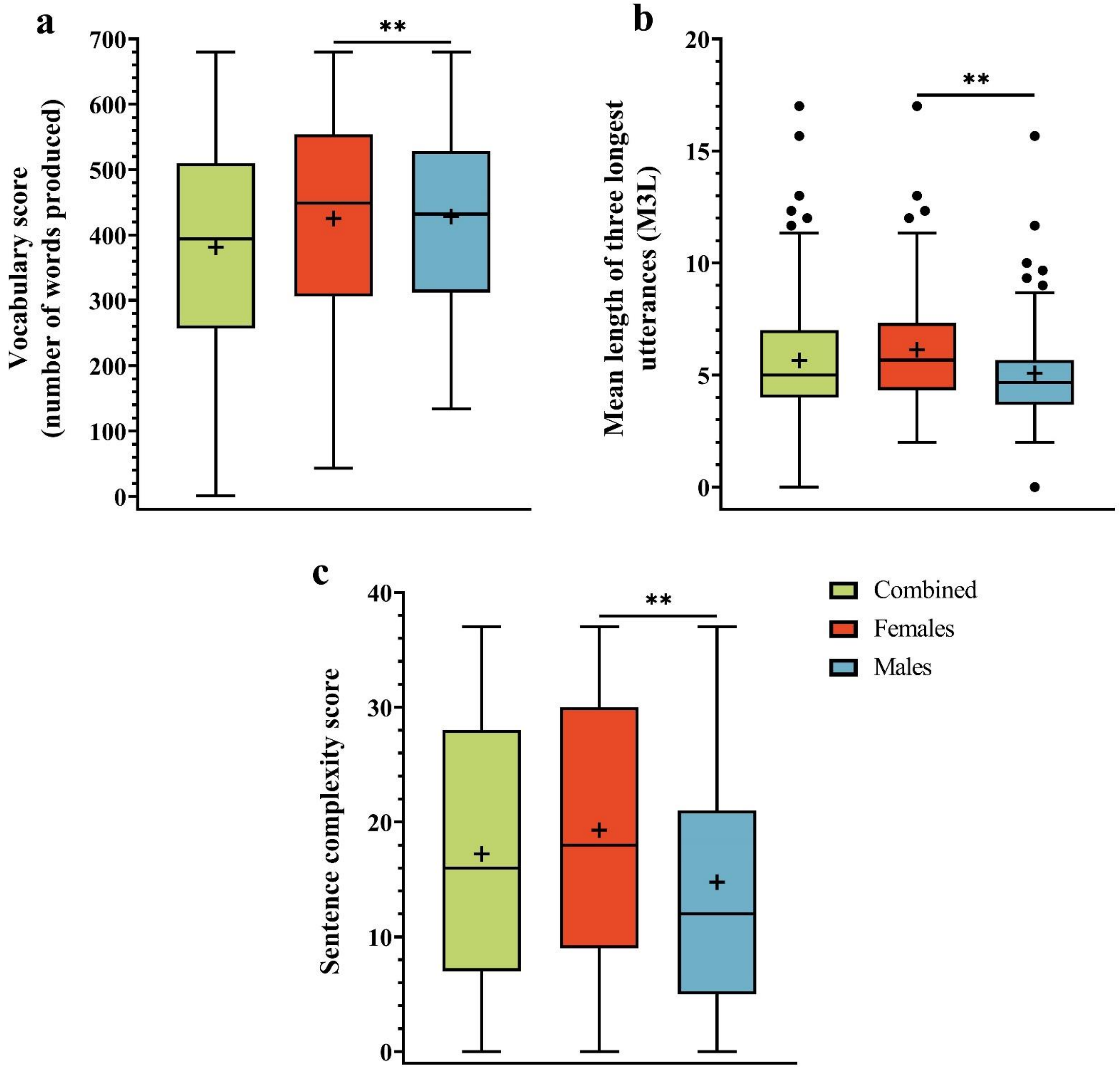

Supplemental Figure S3. Box and whisker plots depicting the range, interquartile range, mean (indicated by +) and median of outcomes on the CDI at 26.5-28.5 months of age in the IKIDS cohort (n = 298). The vocabulary scores for all children in the sample (a) ranged from 1-680 (680 is the maximum score for vocabulary on the CDI). The mean (standard deviation [SD]) vocabulary score for all children in the sample was 381.12 (±169.51), and the median (interquartile range [IQR]) was 394 (250) words produced. On average (SD), females had a larger vocabulary (a) of 425.39 (±153.29) words, with a median (IQR) of 449 (248), compared to males who had a mean (SD) vocabulary size of 335.64 ± 173.79, with a median (IQR) of 329 (267) words ($p < 0.0001$[a]). (b) The range of M3L scores in this sample was 0-17.00, and all children in the sample had a mean (SD) M3L of 5.66 (2.39) with a median (IQR) of 5 (3) morphemes produced. Female children had longer M3L (mean [SD] = 6.14 [±2.50], median [IQR] = 5.67 [3] morphemes produced) than male children (mean (SD) = 5.08 [±2.12], 4.67 [2] morphemes produced; $p = 0.0003$[a]). (c) The range of complexity scores in this sample was 0-37 (37 is the maximum score for complexity on the CDI) with a mean (SD) complexity score for children of 17.23 (11.51) and a median (IQR) of 16 (21). Female children also spoke in more complex sentences (mean [SD] = 19.30 [±11.76], median [IQR] = 18 [21]) than male children (mean [SD] = 14.77 [±10.75], median [IQR] = 12 [16]; $p = 0.003$[a]).

[a]p-values estimated using t-tests
**p < 0.01

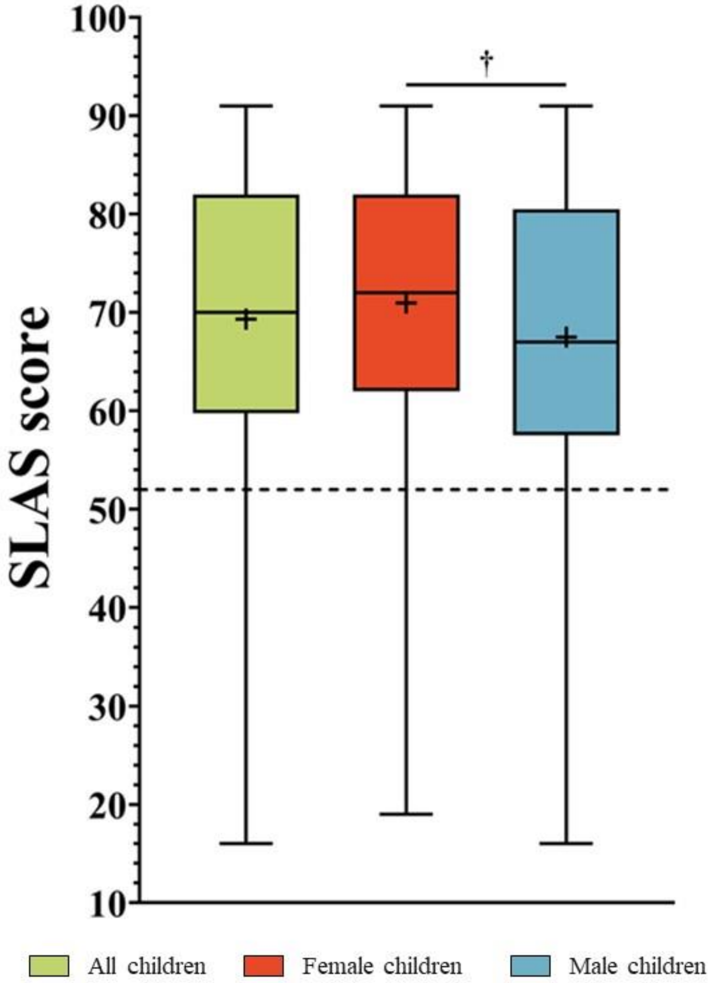

Supplemental Figure S4. Box and whisker plots depicting the range, interquartile range, mean (indicated by +) and median of outcomes on the SLAS total score at 36-38 months of age in the IKIDS cohort (n = 254). Possible SLAS total scores have a range of 13 to 91, and the dotted line indicates the minimum score that would be considered "normal for age" (52). The subset of children with SLAS data available at 36-38 months had scores ranging from 16 to 91 with a mean (standard deviation [SD]) of 69.32 (±15.02), just below the median(interquartile range [IQR]) score of 70 (22). Female children had higher scores (mean[SD] = 70.98 [±14.43], median[IQR] = 72 [20]) compared to male children (mean[SD] = 67.50 [±15.51], median [IQR] = 67 [22]), although the difference was not as dramatic as at 26.5-28.5 months of age (p = 0.07[a]).

[a]p-values estimated using t-tests

[†]$p < 0.10$

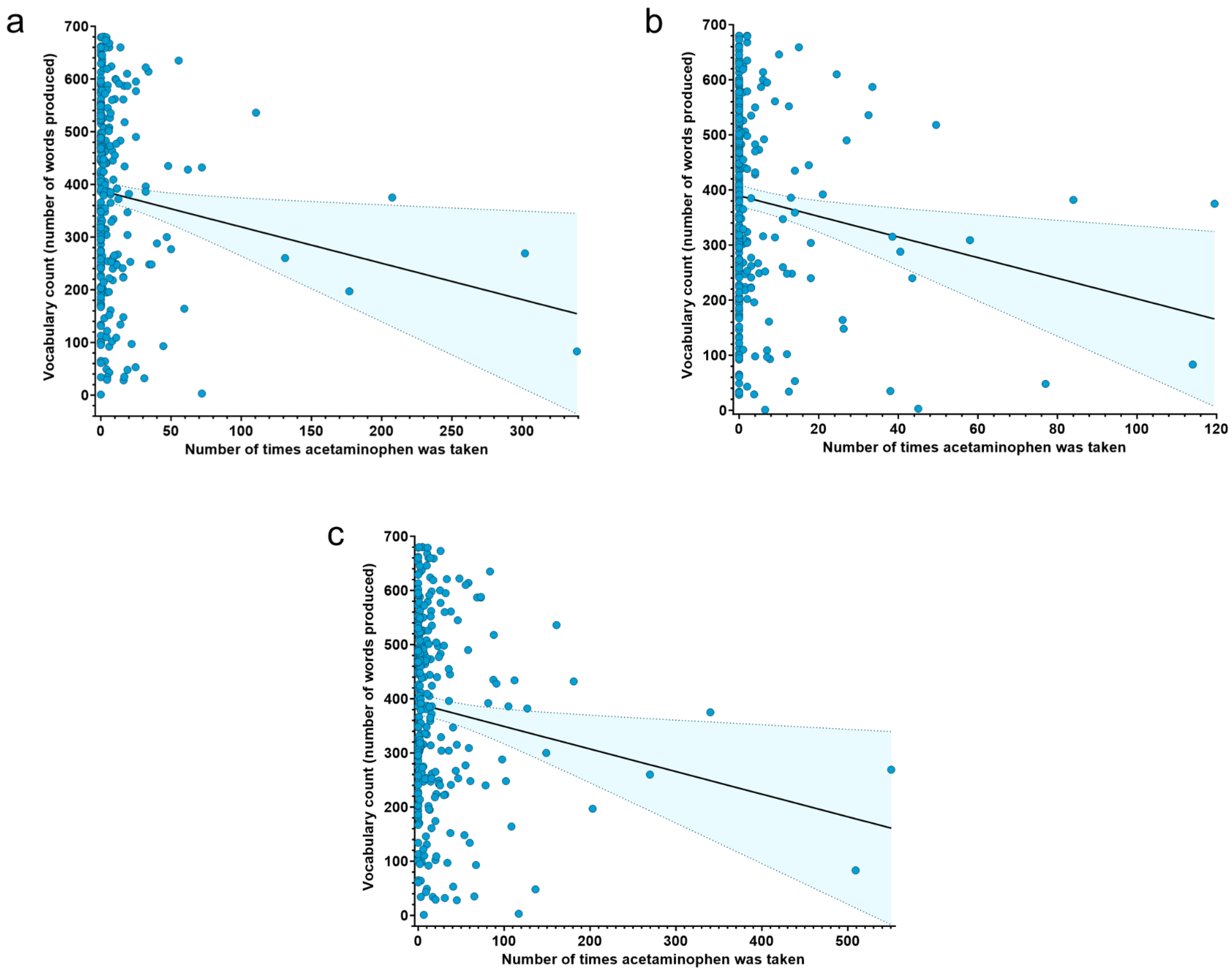

Supplemental Figure S5. Scatter plots with regression lines and 95% confidence intervals of the vocabulary count for all children vs. the number of times acetaminophen was taken during pregnancy associations where p<0.10 are shown, i.e. (a) the second trimester, (b) third trimester, and (c) throughout pregnancy.

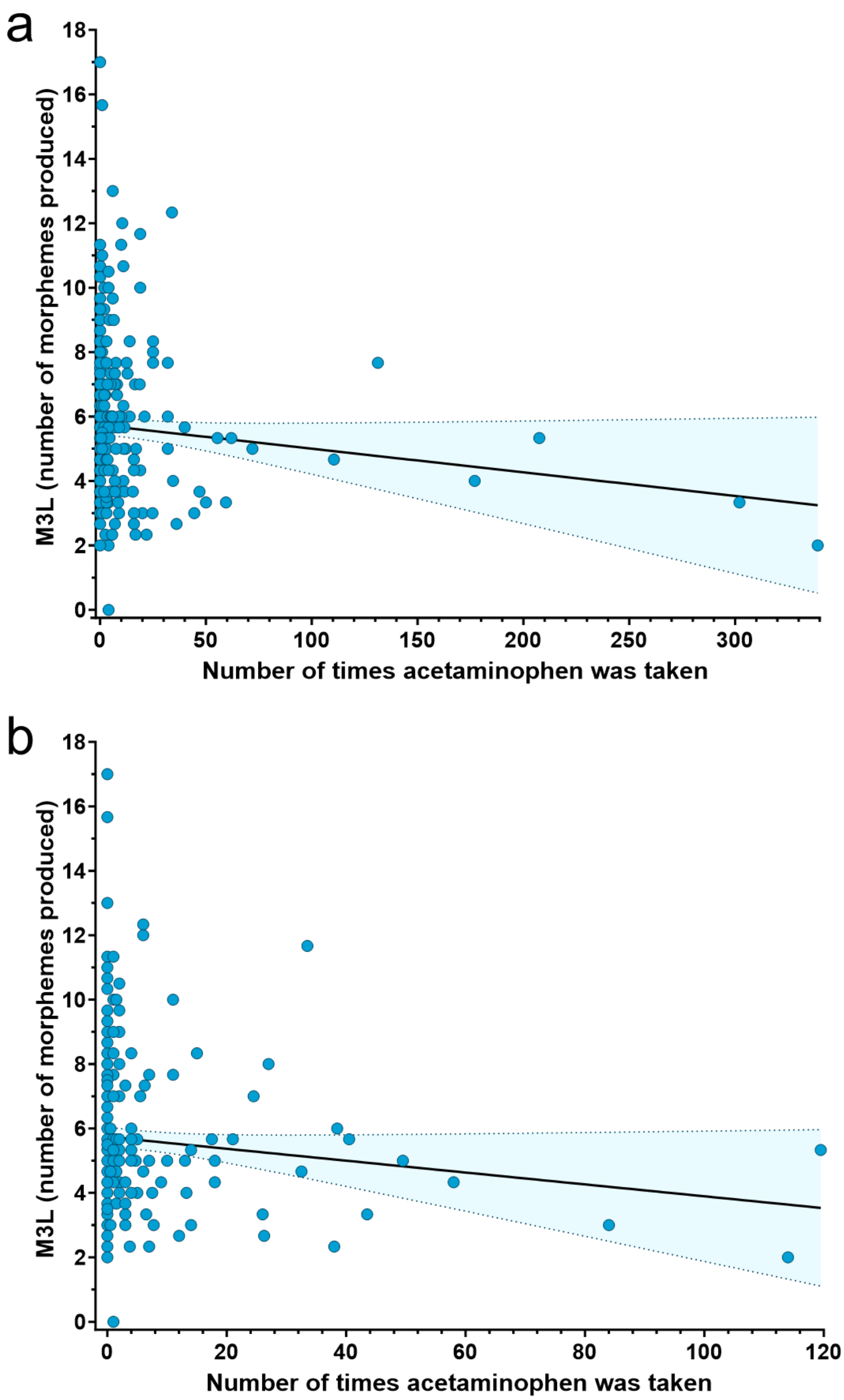

Supplemental Figure S6. Scatter plots with regression lines and 95% confidence intervals of the mean length of utterance for all children vs. the number of times acetaminophen was taken during pregnancy associations where p<0.10 are shown, i.e., (a) the second trimester and (b) third trimester.

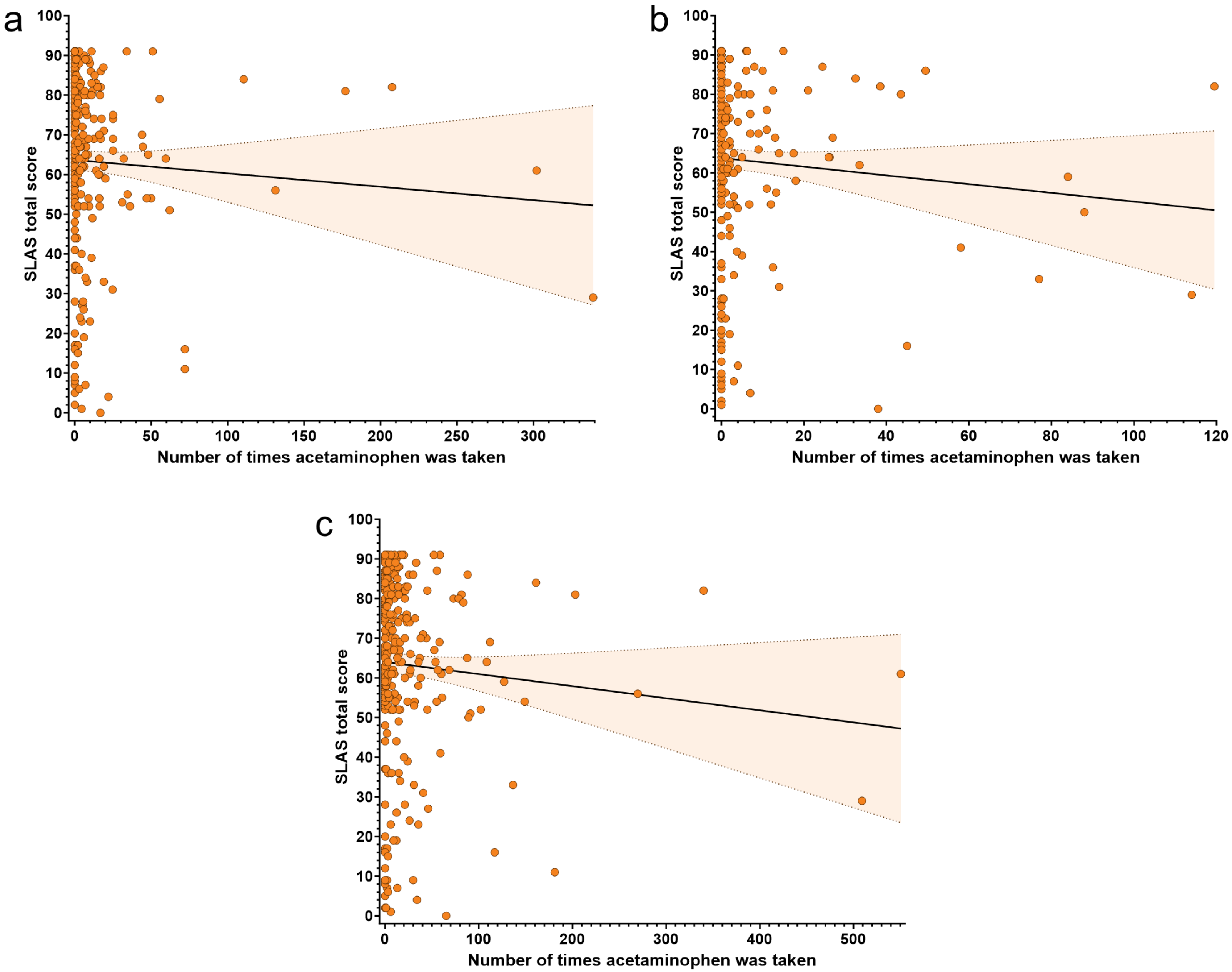

Supplemental Figure S7. Scatter plots with regression lines and 95% confidence intervals of the SLAS total score for all children vs. the number of times acetaminophen was taken during pregnancy associations where $p<0.10$ are shown, i.e. (a) the second trimester, (b) third trimester, and (c) throughout pregnancy.

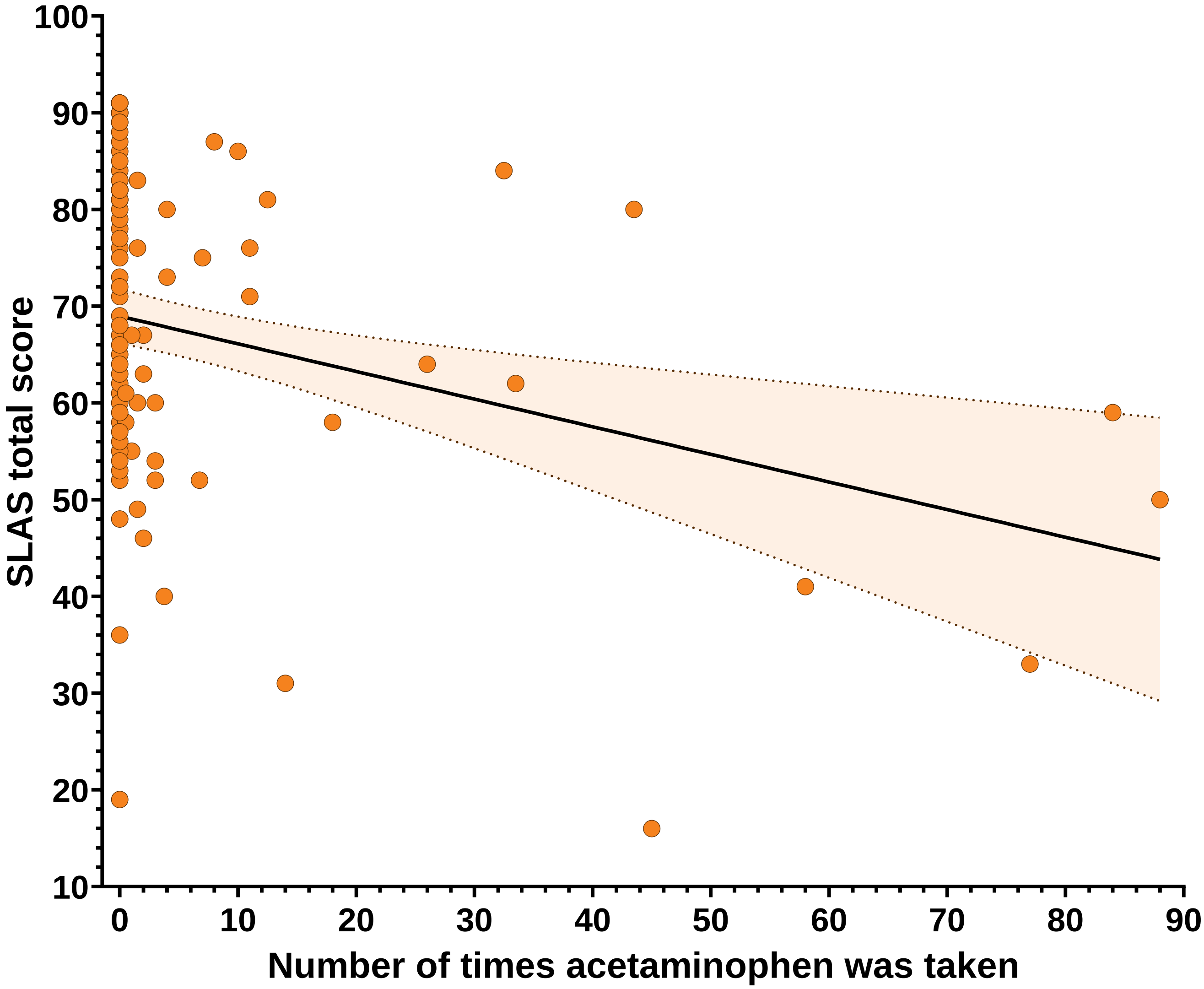

Supplemental Figure S8. Scatter plot with regression line and 95% confidence interval of the SLAS total score for male children vs. the number of times acetaminophen was taken during the third trimester (interaction with sex $p<0.05$, association for male children $p<0.01$ in stratified analysis).

Supplemental Table S2. Results (p-values) of tests comparing demographic characteristics by whether participants reported taking acetaminophen during pregnancy or not within the full sample of children enrolled in IKIDS with exposure data available (n = 532), the subset with CDI data available (n = 298), and the subset with SLAS data available (n = 254).[a]

**Participant Characteristics**	Participants with an infant enrolled in study & exposure data available (n = 532)	Participants who completed the CDI when their child was 26.5-28.5 months (n = 298)	Participants who completed the SLAS when their child was 36-38 months (n = 254)
Birthing parent race & ethnicity	0.05	0.06	0.14
Other parent race & ethnicity	0.05	0.04	0.07
Birthing parent marital status	0.73	0.51	0.76
Birthing parent education	0.23	0.19	0.06
Other parent education	0.18	0.45	0.28
Birthing parent parity	0.37	0.43	0.65
Household income	0.74	0.52	0.93
Birthing parent health insurance status	0.15	--	--
Birthing parent tobacco smoking during 1st trimester	0.89	0.46	0.76
Birthing parent alcohol during 1st trimester consumption	0.80	0.72	0.87
Birthing parent non-native English speaker	<0.0001	0.01	0.10
Child sex	0.83	0.25	0.81
Child race & ethnicity	0.04	0.05	0.12
Delivery type	0.59	0.70	0.20
Birthing parent age (years) at baseline	0.09	0.18	0.38
Birthing parent verbal IQ (PPVT[b] standardized score)	0.83	0.98	0.82
Mean birthing parent stress (PSS-10[c]) score during pregnancy	0.18	0.41	0.27
Mean birthing parent depression (EPDS[d]) score during pregnancy[e]	0.40	0.76	0.62
Child gestational age at birth (weeks)	0.45	0.23	0.19
Child weight at birth (kg)	0.5	0.72	0.29

[a]Chi-square tests were used for categorical variables, t-tests were used for normally distributed continuous variables, and Kruskal-Wallis tests were used for non-normally distributed continuous variables
[b]PPVT-IV: Peabody Picture Vocabulary Test - Fourth Edition
[c]PSS-10: Perceived Stress Scale
[d]EPDS: Edinburgh Postnatal Depression Scale
[e]Kruskal-Wallis test was used due to skewness of EPDS scores

Supplemental Table S3. Frequency of participants who reported the same indications as the those provided for acetaminophen (yes/no) for another analgesic by whether they took acetaminophen or not for those with data available at 26.5-28.5 and 36-38 months.

	**Participants who completed the MB-CDI when their child was 26.5-28.5 months**		**Participants who completed the SLAS when their child was 36-38 months**	
**Indication**	Participant took another analgesic during pregnancy (n = 90)	Participant took acetaminophen during pregnancy (n = 208)	Participant took another analgesic during pregnancy (n = 80)	Participant took acetaminophen during pregnancy (n = 174)
Pain	5 (5.6)	14 (6.7)	4 (5.0)	11 (6.3)
Illness	1 (11.1)	5 (2.4)	1 (1.3)	3 (1.7)
Other	9 (10.0)	20 (9.6)	7 (8.8)	15 (8.6)

Supplemental Table S4. Results of Wilcoxon-Rank Sum Tests for comparison of acetaminophen use during pregnancy among the full sample of children enrolled in IKIDS (n = 535), the subset with CDI data available (n = 298), and the subset with SLAS data available (n = 254).

Exposure	Comparison	p-value
1st Trimester	Entire sample vs. Subset with CDI data available	0.89
	Entire sample vs. Subset with SLAS data available	0.77
	Subset with CDI data available vs. Subset with SLAS data available	0.57
2nd Trimester	Entire sample vs. Subset with CDI data available	0.52
	Entire sample vs. Subset with SLAS data available	0.12
	Subset with CDI data available vs. Subset with SLAS data available	0.69
3rd Trimester	Entire sample vs. Subset with CDI data available	0.79
	Entire sample vs. Subset with SLAS data available	0.92
	Subset with CDI data available vs. Subset with SLAS data available	0.98
Entire pregnancy	Entire sample vs. Subset with CDI data available	0.64
	Entire sample vs. Subset with SLAS data available	0.007
	Subset with CDI data available vs. Subset with SLAS data available	0.14

Supplemental Table S5. Distributions and Pearson correlations for CDI scores (n = 298) and SLAS scores (n = 254) in IKIDS for all children and by sex.

	Distributions of outcome measures			Pearson correlations among outcome measures		
All children						
**Outome measure**	**n**	**Mean ± SEM**	**Median**	**Vocabulary score**	**M3L**	**Sentence complexity score**
Vocabulary score (number of words produced)	298	381.12 ± 9.82	394	--	--	--
Mean length of longest utterances (M3L)	256	5.66 ± 0.15	5.00	0.69	--	--
Sentence complexity score	234	17.23 ± 0.75	16.00	0.80	0.71	--
SLAS total score	254	69.32 ± 0.94	70.00	0.61	0.44	0.53
Female children						
Vocabulary score (number of words produced)	151	425.39 ± 12.47	449.00	--	--	--
Mean length of longest utterances (M3L)	140	6.14 ± 0.21	5.67	0.66	--	--
Sentence complexity score	127	19.30 ± 1.04	18.00	0.78	0.69	--
SLAS total score	133	70.98 ± 1.25	72.00	0.67	0.50	0.56
Male children						
Vocabulary score (number of words produced)	147	335.64 ± 14.33	329	--	--	--
Mean length of longest utterances (M3L)	116	5.08 ± 0.20	4.67	0.69	--	--
Sentence complexity score	107	14.77 ± 1.04	12.00	0.81	0.71	--
SLAS total score	121	67.50 ± 1.41	67.00	0.54	0.31	0.48
